# MGMT promoter methylation status for glioblastoma: defining the clinically relevant cut-off value for pyrosequencing

**DOI:** 10.1007/s11060-026-05633-0

**Published:** 2026-05-20

**Authors:** Nathalie Skarin, Martin Hallbeck, Katja Werlenius, Zdenek Rohan, Maria Sandström, Eszter Turanyi, David Löfgren, Håkan Johansson, Michael Strandéus, Björn Tavelin, Annika Malmström

**Affiliations:** 1 Department of Oncology, County Hospital Kalmar, Kalmar, Sweden; 2 Department of Pathology, University Hospital Linköping, Linköping, Sweden; 3 Department of Biomedical and Clinical Sciences, Linköping University, Linköping, Sweden; 4 Department of Oncology, Sahlgrenska University Hospital, Gothenburg, Sweden; 5 Department of Oncology, Institute of Clinical Sciences, Sahlgrenska Academy, University of Gothenburg, Gothenburg, Sweden; 6 Department of Medical Biosciences, Umeå University, Umeå, Sweden; 7 Department of Diagnostics and Interventions, Oncology, Umeå University, Umeå, Sweden; 8 Department of Pathology, University Hospital Örebro, Örebro, Sweden; 9 Department of Oncology, Faculty of Medicine & Health, University Hospital Örebro, Örebro, Sweden; 10 Department of Research, Region Kalmar County, Kalmar, Sweden; 11 Department of Oncology, County Hospital Ryhov, Jönköping, Sweden; 12 Clinical Research Unit, Cancercentrum, Region Västerbotten, Umeå University Hospital, Umeå, Sweden; 13 Clinical Department of Geriatrics and Palliative Medicine in Linköping, Region Östergötland, Linköping, Sweden

**Keywords:** Glioblastoma, MGMT promoter methylation cut-off, Pyrosequencing, Survival, Prognostic factors, Treatment prediction

## Abstract

**Purpose:**

MGMT promoter methylation is a key predictive biomarker for response to alkylating agents in glioblastoma. However, there is no consensus regarding optimal analytical method or cut-off. This study aimed to define a clinically relevant survival-based cut-off value for MGMT using a standardized pyrosequencing assay.

**Methods:**

Patients from five Swedish university hospitals with MGMT promoter methylation status analyzed using the Therascreen MGMT Pyro Kit, investigating CpGs 76–79, were identified. Glioblastoma patients treated with radiotherapy and concomitant temozolomide were selected from the Swedish CNS Tumor Registry. Quantitative MGMT status, both mean value and percentage of methylation for each individual CpG, was analyzed using an unsupervised bimodal normal mixture model and a survival-informed approach adjusted for established prognostic factors.

**Results:**

A total of 451 patients were included. The unsupervised model identified a cut-off at ≥ 11% for methylated MGMT, whereas the supervised, survival-informed analysis defined a cut-off for unmethylated tumors at ≤ 8%. A small intermediate gray zone (> 8% to < 11%) was observed. No individual CpG provided superior predictive performance compared to the mean of all CpGs.

**Conclusion:**

A clinically relevant, survival informed three-tier classification of MGMT promoter methylation is proposed for the pyrosequencing kit analyzing CpGs 76–79 in glioblastoma. A threshold of ≤ 8% identifies a subgroup with truly unmethylated tumors, while ≥ 11% defines a clearly methylated group. The intermediate range represents a gray zone. These cut-offs support individualized therapy by improving clinically relevant stratification and facilitate future clinical trials focusing on patients with truly unmethylated MGMT.

**Supplementary Information:**

The online version contains supplementary material available at 10.1007/s11060-026-05633-0.

## Introduction

Glioblastoma (GBM) is the most common and aggressive primary malignant brain tumor in adults [1]. It has poor prognosis and limited treatment options [2, 3]. Diagnostic and prognostic evaluations regarding GBM have been gradually refined by the implementation of molecular biomarkers, one being the methylation status of the *O6-methylguanine-DNA methyltransferase* (*MGMT*) gene promoter. Methylation of cytosine-phosphate-guanine (CpG) islands at specific CpG sites within the *MGMT* promoter silences the gene. This results in decreased production of the DNA repair enzyme MGMT, thereby leading to DNA alkylation caused by chemotherapy remaining unrepaired and cytotoxic cell death in tumor cells [4]. Methylation of the *MGMT* promoter has been shown to correlate with increased survival in GBM patients treated with alkylating agents, such as temozolomide (TMZ), making it the sole positive predictive factor available [5–9].

Standard treatment for GBM since 2005 is maximum safe resection, postoperative radiotherapy with concomitant TMZ (RT/TMZ) followed by six cycles of TMZ. For patients treated according to this protocol, median overall survival (OS) in clinical studies has been shown to range between 12.6 and 14.5 months in patients with unmethylated *MGMT* promoter (uMGMT), compared to 23.4–32.1 months, in patients with methylated *MGMT* promoter (mMGMT) [2, 10–12]. Median OS for all newly diagnosed patients with GBM in Sweden during the period 2009–2024 was 11.8 months, according to the Swedish Brain Tumor Registry (SBTR; covering 1999–2017) and the Swedish CNS Tumor Registry (SCNSTR; covering 2018 and onward) [13].

*MGMT* promoter methylation (MGMTpm) status can be assessed using different methods. To this date, there is no consensus regarding the optimal method for MGMTpm analysis, or optimal cut-off for the same method [14, 15]. Some have reported bimodal distribution for MGMTpm values and proposed the cut-off at the minimum value between the two distributions [16]. Dunn examined 12 CpGs (CpG 73–84) and proposed the cut-off ≥ 10% for mMGMT, based on the mean MGMTpm of normal brain samples +/- two standard deviations [17]. A third option is to establish a cut-off based on outcome, such as in the study by Quillien where the Pyromark MGMT kit investigating CpGs 74–78 in exon 1 of the *MGMT* gene by pyrosequencing was used and a cut-off of ≥ 9% for mMGMT was proposed [18]. A subsequent study by Quillien to validate the clinical Therascreen kit, which analyses CpGs 76–79, shows that the Pyromark and Therascreen assays can be used interchangeably with the same cut-offs, with 95% of patients identically classified with the two assays if using the cut-off 8% and 97% if applying the cut-off 12% [19].

Since 2014, several departments of pathology in Sweden investigate MGMTpm status using Therascreen. The result is presented as the average percentage of methylation of the 4 CpGs. At the departments in this study, some used the cut-off < 10% for uMGMT, 10–25% for methylated and > 25% for highly methylated tumor, based on two studies [17, 20]. Others deemed samples with a methylation ≥ 9% as methylated, based on the study by Quillien [18].

Hegi determined a *clinically* relevant cut-off, and a gray zone, taking survival outcome and prognostic factors into account, for MGMTpm status investigated with methylation specific PCR (msPCR). In the gray zone a fraction of patients are expected to respond to TMZ treatment, whereas patients identified with truly uMGMT tumor seem to derive no benefit [21]. These results were confirmed when applied to an elderly patient population [22]. We aimed to determine the corresponding clinically relevant cut-off for pyrosequencing, the technique used in Sweden, and many other countries.

## Materials and methods

### Data selection

During the study period, MGMTpm status was analyzed using the Therascreen MGMT Pyro Kit at the pathology departments of all seven Swedish university hospitals performing surgery for brain tumors. Five of these centers contributed patients to the study. For one participating center, MGMTpm analysis was performed at one of the other participating university hospitals. The Therascreen kit analyzes CpG sites 76–79 in chr10:129,467,255 − 129,467,273 (Reference genome: GRCh38.p14). Analysis was performed as part of standard clinical diagnostic work-up, on tumor enriched, macrodissected, formalin-fixed, paraffin embedded tissue according to the manufacturer’s instructions. Data was obtained on patients whose tumors were analyzed using the Therascreen assay to determine MGMTpm status between 1st of January 2014 up to 31st of December 2022, allowing for follow-up. Additional data were retrieved from the SCNSTR, on histopathological diagnosis, IDH status, extent of resection, oncological treatment, preoperative performance status measured by the Eastern Cooperative Oncology Group (ECOG) performance status scale (preopPS), and survival. Extent of resection was primarily determined from postoperative MRI. In cases where imaging assessment was unavailable or inconclusive, mainly biopsy patients, surgical intent was used. Oncological treatment data in the SCNSTR were available from December 2017 and patients with MGMT data before that were therefore excluded from the analysis. MGMTpm status for each individual CpG and the mean was provided by the pathology departments.

Patients diagnosed histologically with GBM, IDH-wildtype (IDHwt) (*n* = 451), that received RT/TMZ according to the studies by Stupp [2] or Perry [5], and with available information regarding quantitative MGMTpm status, were included in the final analysis.

### Statistical analysis

To determine the cut-off for MGMTpm, we assumed that the patients’ tumors can be classified as either methylated or unmethylated. For both the mean MGMTpm status and the status for each individual CpG, we determined an unsupervised cut-off using a bimodal normal mixture model (BNMM) fitted to the logarithmized MGMTpm status, with the cut-off defined as the intersection point of the two component distributions. We determined a supervised, survival-informed, cut-off using an accelerated failure time (AFT) model with a log-logistic distribution [23]. An AFT model was chosen to allow direct comparison of survival time ratios and optimize threshold discrimination. The model included dichotomized methylation status (methylated vs. unmethylated) adjusted for age, extent of surgery, and preopPS. Candidate cut-offs (between 1 and 25% with 1% increments) were evaluated by refitting the model with 5-fold cross-validation, and the optimal threshold was selected based on the highest concordance index. The concordance index reflects the proportion of patient pairs for which predicted and observed survival times are concordant [24]. The interval between the unsupervised and survival-informed cut-offs was defined as a gray zone. OS was defined as the time from date of primary surgery to death from any cause; patients alive at last follow-up were censored. To test whether survival differed between patients with methylated, unmethylated, and gray zone MGMTpm status, we performed log-rank test and Kaplan-Meier analysis. The proposed cut-offs for the mean and for each individual CpG were compared by highest concordance index. All analyses were performed using R statistical computing environment (R Core Team, Vienna, Austria), version. 4.4.3 [25]. The BNMM was fitted using the mixtools package (v. 2.0.0.1) [26] and the AFT model using the survival package (v. 3.8-3) [23, 27]. Results were considered statistically significant at *p* < 0.05. For additional statistical information, see Supplement.

## Results

### Study population

**Fig. 1:**
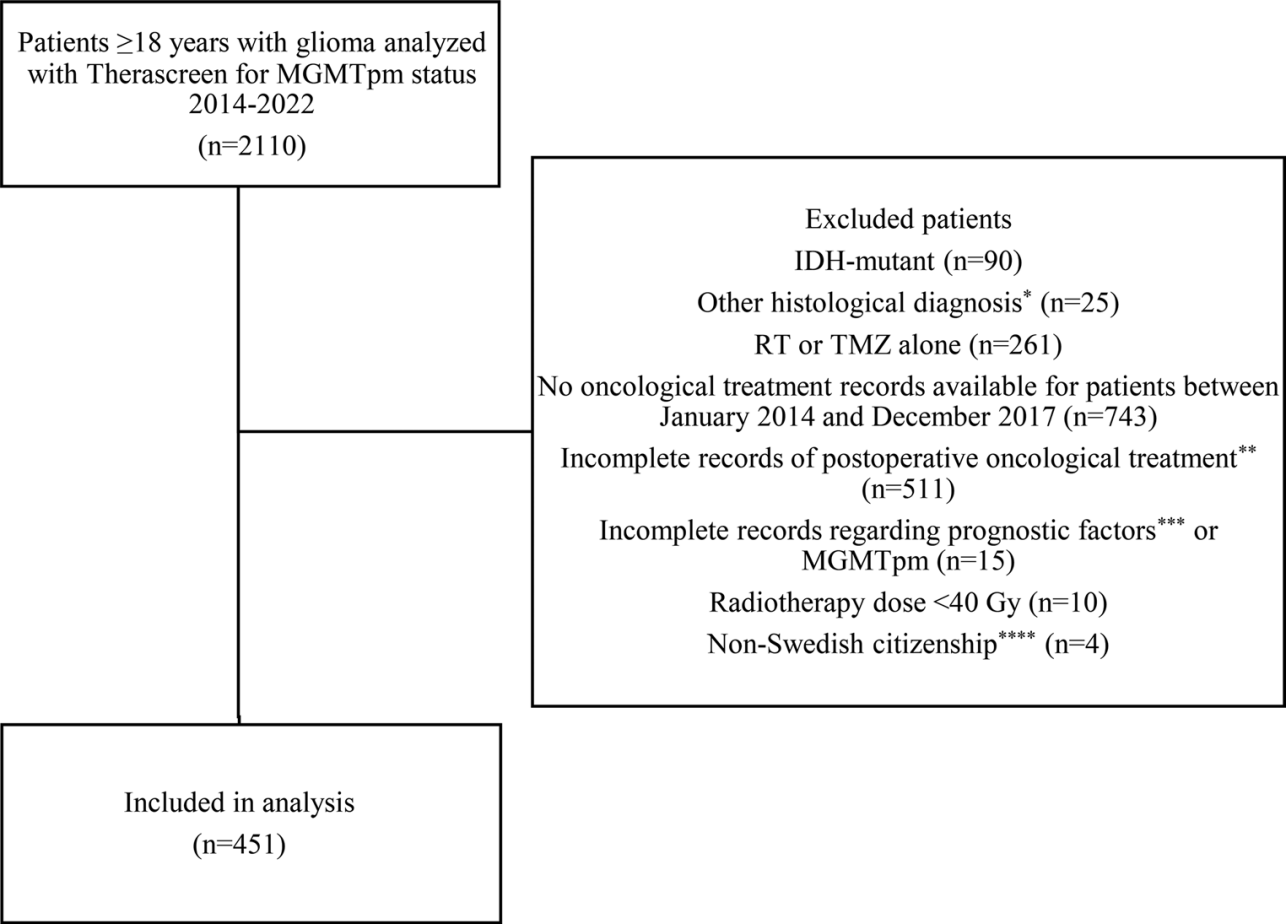
Flowchart of patient selection. MGMTpm, MGMT promoter methylation; RT, radiotherapy; TMZ, Temozolomide. ^*^Meningioma, ependymoma, diffuse midline glioma, pilocytic astrocytoma, ganglioglioma, fibrillary astrocytoma, oligodendroglioma IDHmut, gemistocytic astrocytoma IDHmut and germinoma ^**^Including patients not receiving postoperative treatment. ^***^ preopPS, or extent of resection ^****^ thereby absent from the SQR

Figure 1 delineates a flowchart of patient inclusion and exclusion. We identified 2110 patients, ≥ 18 years with MGMTpm status analyzed by the pyrosequencing kit between 2014 and 2022.

In total, 451 patients were included in the final analysis, while 1659 patients were excluded due to reasons as described in Fig. 1. Postoperative MRI assessment was available for 82.5% of patients (*n* = 372). For the remaining 17.5% (*n* = 79), extent of resection was classified based on surgical intent; with the majority representing biopsy (*n* = 68).

Of the included 451 patients, 109 patients received hypofractionated RT to 40 Gy [8]. The remaining 355 patients received ≥ 50 Gy, with all but nine receiving 60 Gy [2]. Baseline characteristics were balanced between the uMGMT and mMGMT groups, but not in the gray zone cohort, where worse prognostic factors - including older age, less extensive resection and higher preopPS - were observed. Similarly, a lower proportion of patients in the gray zone received 60 Gy (53.3%), compared to patients with mMGMT (75.5%) and uMGMT (75.0%). Patient characteristics are summarized in Table 1.

**Table 1:** Patient demographics and clinical characteristics

Variable	Total (*n* = 451)	Methylated MGMT (*n* = 188)	Gray zone (*n* = 15)	Truly unmethylated MGMT (*n* = 248)
Age, years				
Median (range)	62 (18–82)	63 (18–82)	66 (33–77)	62 (30–81)
Sex, n (%)				
Male	292 (64.7%)	114 (60.6%)	8 (53.3%)	170 (68.5%)
Female	159 (35.3%)	74 (39.4%)	7 (46.7%)	78 (31.5%)
preopPS^a^, n (%)				
0	116 (25.7%)	47 (25.0%)	2 (13.3%)	67 (27.0%)
1	158 (35.0%)	67 (35.6%)	4 (26.7%)	87 (35.1%)
2	159 (35.3%)	64 (34.0%)	7 (46.6%)	88 (35.5%)
3	14 (3.1%)	8 (4.3%)	1 (6.7%)	5 (2.0%)
4	4 (0.9%)	2 (1.1%)	1 (6.7%)	1 (0.4%)
Extent of resection^b^, n (%)				
Gross total resection	162 (35.9%)	70 (37.2%)	2 (13.3%)	90 (36.3%)
Near-total resection (< 1 cm³ residual tumor)	105 (23.3%)	37 (19.7%)	3 (20.0%)	65 (26.2%)
Subtotal resection	116 (25.7%)	54 (28.7%)	6 (40.0%)	56 (22.6%)
Biopsy	68 (15.1%)	27 (14.4%)	4 (26.7%)	37 (14.9%)
Total radiotherapy dose received, n (%)				
40–41 Gy	106 (23.5%)	43 (22.9%)	7 (46.7%)	56 (22.6%)^c^
50-<60 Gy	9 (2.0%)	3 (1.6%)	0 (0%)	6 (2.4%)
60 Gy	336 (74.5%)	142 (75.5%)	8 (53.3%)	186 (75.0%)

^a^ Preoperative performance status measured by the ECOG performance status scale. ^b^ Extent of resection was primarily determined based on postoperative MRI. When imaging was unavailable or inconclusive (17.5%, *n* = 79), classification was based on surgical intent; most cases were biopsy patients (*n* = 68). ^c^Two of these patients started treatment according to the standard RT/TMZ regimen with 2 Gy fractions, but due to early deterioration and large tumor volumes their treatment was altered to hypofractionated RT after three fractions

**Fig. 2:**
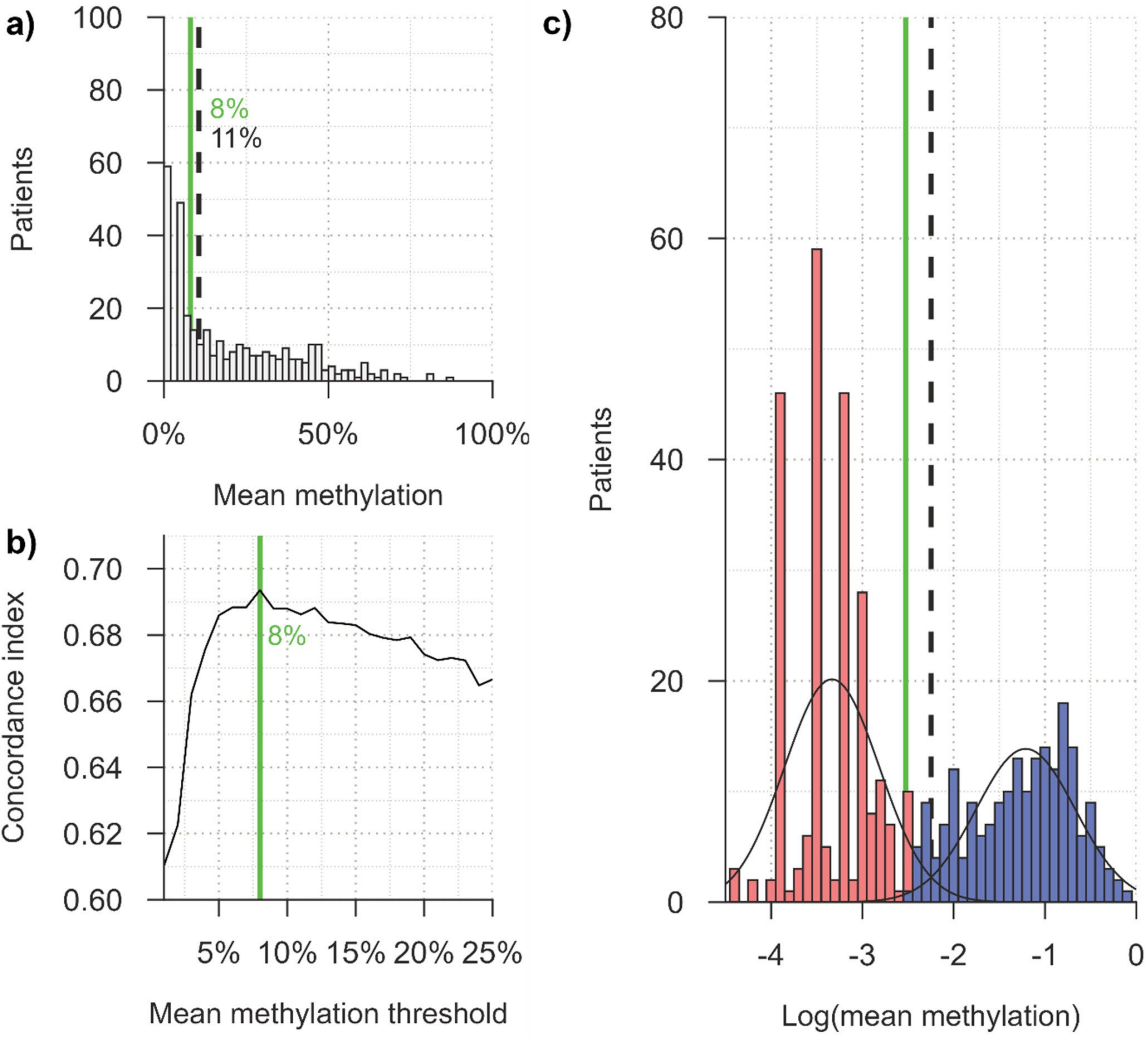
*MGMT* promoter methylation status and cut-offs. (**a**) Distribution of mean MGMTpm status of all 451 patients. The solid green line representing the supervised cut-off (**b**) and the dashed black line representing the unsupervised cut-off (**c**). Distributions for each individual CpG are presented in Supplementary Fig. 1. (**b**) Cut-off supervised by OS and adjusted for prognostic factors: age, extent of resection and preoperative ECOG performance status (solid green line), determined through 5-fold cross-validation with the highest concordance index corresponding to the best performing AFT (accelerated failure time) model. (**c**) Bimodal normal mixture model, with unsupervised cut-off (dashed black line) determined as the intersection point of the two distributions. Blue bars represent patients with methylated MGMT and red bars represent patients with unmethylated MGMT, according to the supervised cut-off (solid green line, **b**)

### *MGMT* promoter methylation cut-offs and survival analysis

The distribution of mean tumor *MGMT* methylation for each included patient is presented in Fig. 2a. The unsupervised cut-off for mMGMT was estimated to ≥ 11%, determined by the BNMM as the intersection point between the two logarithmized MGMTpm distributions (Fig. 2c). The supervised cut-off for uMGMT, derived by including OS and adjusted for prognostic factors, was ≤ 8%, corresponding to the highest concordance index in the best performing AFT model (Fig. 2b; see Supplementary Table 2 for the parameter estimates). The interval between the unsupervised and survival-informed thresholds was defined as the gray zone (Fig. 2c).

In addition, methylation levels at individual CpG sites were evaluated using the BNMM and AFT models. Distribution of methylation of each individual CpG is presented in Supplementary Fig. 1, with BNMM results in Supplementary Fig. 2 and AFT results in Supplementary Fig. 3. No individual CpG demonstrated superior predictive performance compared to mean methylation, although differences were small (concordance index differences: -2.13–0.21%; Supplementary Fig. 3).

At the time of analysis (20 June 2025), 7.7% (*n* = 35) of patients were still alive. Using the supervised cut-off (≤ 8%), 55.0% (*n* = 248) were classified as truly unmethylated. Employing the unsupervised cut-off (≥ 11%), 41.7% (*n* = 188) were classified as methylated, while 3.3% (*n* = 15) fell within the gray zone (> 8 to < 11%). The ≤ 8% cut-off identified 99% of tumors classified as methylated by the BNMM approach.

**Fig. 3:**
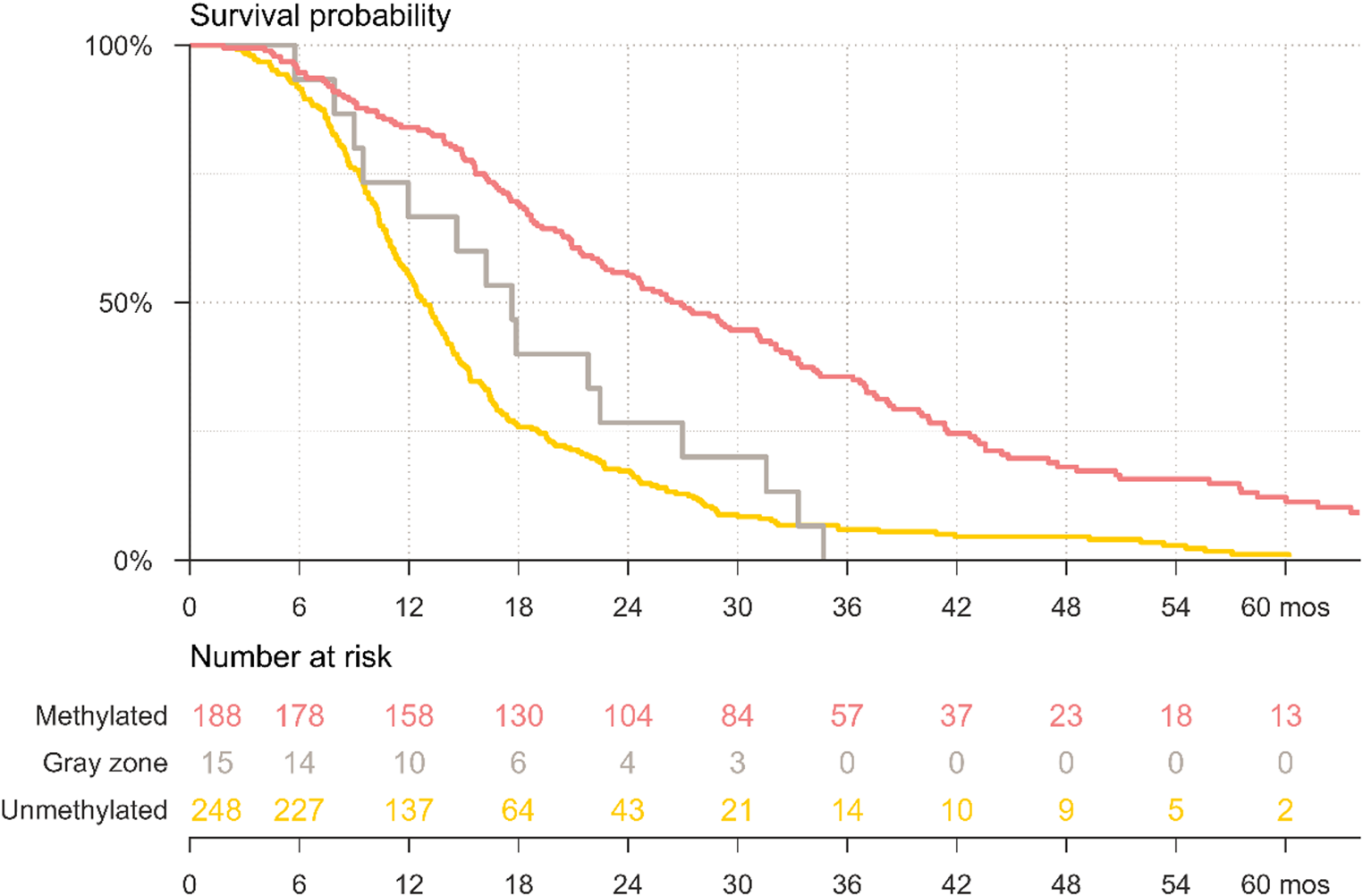
Kaplan-Meier curves demonstrating survival for patients with truly uMGMT, MGMTpm in the gray zone and mMGMT GBM

Median OS was 12.9 months in the uMGMT group, 17.6 months in the gray zone, and 26.6 months in the mMGMT group (Fig. 3). OS differed significantly between uMGMT and mMGMT (log-rank test, *p* < 0.001), uMGMT and gray zone (log-rank test, *p* < 0.001), and mMGMT and gray zone (log-rank test, *p* = 0.001). Survival rates for the three groups at 12 months were 55.2%, 66.7% and 84.0%, at 24 months 17.3%, 26.7% and 55.3%, and at 60 months 0.8%, 0% and 6.9%, respectively (Supplementary Table 1). For survival in relation to each CpG see Supplementary Fig 4.

## Discussion

The clinically relevant cut-off value for MGMTpm status in GBM remains an important and unresolved issue in routine clinical practice when guiding the use of alkylating agents such as TMZ. The 2023 EANO guideline recommends assay-specific validation of MGMT testing, including the definition of cut-offs and potential gray zones [14]. Variability in analytical methods and thresholds continues to create uncertainty in individualized treatment recommendations and limits comparability between clinical studies.

In this population-based cohort of 451 GBM patients treated with concomitant RT/TMZ, MGMTpm was assessed in routine clinical practice using pyrosequencing of CpGs 76–79. Using a survival-informed threshold (≤ 8%), 55.0% of tumors were classified as truly unmethylated, while 41.7% were classified as methylated using an unsupervised threshold (≥ 11%), leaving 3.3% within an intermediate gray zone. Median overall survival was 12.9, 17.6, and 26.6 months, respectively. These findings support a clinically relevant three-tier classification of MGMTpm.

Our results align closely with previously proposed cut-off values for MGMTpm determined by pyrosequencing. A prior cohort of 139 patients proposed a similar three-tier system (0–8%, 9–12%, ≥ 13%) using the PyroMark assay supervised for survival but without adjustments for prognostic factors [18].

Quillien aimed to determine which individual CpG and/or combination of CpGs best predict outcome [28]. Investigating 16 CpGs (CpGs 74–89) analyzed by pyrosequencing in 89 GBM patients, they concluded that CpG 89, means of CpGs 84–88, 85–89 and 74–89 correlated strongest with survival. Chai investigated the predictive values of individual CpGs between 75 and 82 as well as combinations of these CpGs, including the combination 76–79 used in our cohort. They determined that it is sufficient to look at four consecutive CpG sites to determine MGMTpm status, e.g. sites 76–79, with equivalent robustness as if investigating more CpGs. Poorer predictive value was shown when looking at fewer than four CpGs [29]. In accordance with this, our results show that the mean of CpGs 76–79 is superior compared to any single included CpG.

Previous work by Hegi demonstrated that clinically relevant cut-offs for MGMTpm status with msPCR can be determined using survival- and prognostic factor-adjusted approaches [21, 22]. For elderly patients, applying the appropriate cut-off when using msPCR demonstrated that it is safe to omit TMZ in cases with unmethylated tumors, with no benefit of addition of TMZ to RT, and worse outcome for these patients when treated with single modality TMZ compared to RT [22]. In line with these studies, we incorporated survival and key prognostic factors when deriving cut-offs for pyrosequencing of CpGs 76–79. We found that 55% had uMGMT tumor, in contrast to Hegi´s results of 43.1% [22]. In Hegi´s study several cohorts were included, these having fractions of uMGMT between 36.6% and 51.9%, the upper range being close to our findings. Also, in published cohorts approximately 55% are commonly reported as uMGMT [30, 31].

Our results extend previous observations by supporting a three-tier classification in a large population-based dataset and indicate that a cut-off at ≤ 8% improves identification of patients with truly unmethylated tumors. Importantly, this threshold captured 99% of patients classified as mMGMT by the unsupervised model, minimizing the risk of withholding potentially effective alkylating agent treatment.

Patients with truly unmethylated tumors are not expected to benefit from TMZ as MGMT status has been shown to be predictive for response to alkylating agents in IDHwt tumors, whereas it has been identified as prognostic for IDHmut gliomas [5, 31, 32]. The proposed threshold may help inform decisions regarding omission of TMZ in patients unlikely to benefit, thereby reducing unnecessary toxicity and hospital visits. This could potentially improve quality of life and allow patients to make better use of their survival time. It would also improve health resource utilization. However, treatment decisions should remain individualized, and prospective validation could be of further value when such thresholds are used to guide omission of therapy in routine clinical practice.

Although relatively few patients had MGMT results within the gray zone, these findings remain clinically relevant for individualized treatment decision-making. Patients with MGMTpm in the gray zone fit for concurrent RT/TMZ, should be recommended standard treatment to potentially benefit from TMZ. In older or frail patients, treatment decisions require individualized consideration, incorporating clinical factors as well as patient preferences. One could argue in favour of single TMZ due to the findings in the Nordic trial [6] where patients who received TMZ had improved QoL at follow-up, compared to RT only, where QoL instead declined. Accordingly, the potential implications of gray zone MGMTpm status should be included in personalized treatment recommendations in cases were both RT and TMZ are valid options.

Younger, fit patients with uMGMT GBM constitute a subgroup who, according to our and the studies by Hegi [21, 22] are unlikely to benefit from TMZ and have an unmet need for improved treatment strategies. The proposed lower cut-off at 8% could therefore be useful for refining patient selection in future clinical trials, particularly to identify patients with GBM who may be candidates for alternative first-line strategies where TMZ is omitted.

In GBM intratumoral variability in MGMTpm status has been reported, especially in case of biopsy only. Research by Gempt analyzing multi-biopsy samples from 56 patients demonstrated concordant methylation status in as high as 84% of cases [33]. For a minority, especially patients evaluated on a single biopsy, the results of MGMT analysis may not fully reflect the tumor’s methylation landscape and should therefore be interpreted with caution when guiding clinical decisions.

One limitation of our study is the small number of patients in the gray zone. Despite this, we observed significant survival differences between methylation groups, even though patients in the gray zone exhibited less favorable prognostic characteristics compared to both the methylated and unmethylated groups, and a smaller proportion receiving standard 60 Gy (Table 1). Pyrosequencing has been reported to classify tumor MGMT status slightly more accurately than msPCR [34–36] which may explain the smaller gray zone in our cohort (3.3%) compared to that reported by Hegi (10.5%).

Missing treatment data in the Swedish CNS Tumor Registry was primarily due to their unavailability in early cohorts with MGMTpm analyzed in 2014–2017. A proportion of excluded patients with missing records from December 2017 and onward are expected not to have received any postoperative oncological treatment, this information not being captured in the registry. While incomplete reporting cannot be fully excluded as a source of bias, this is unlikely to have introduced systematic bias affecting the study outcome.

A key strength of our study is the inclusion of a larger, nationwide cohort compared to previous studies evaluating MGMTpm by pyrosequencing. Importantly, the proposed cut-off and reported survival outcomes are derived from real-world data rather than selected trial populations. Furthermore, only patients with GBM defined according to the most recent WHO classification (i.e., IDHwt) were included. This distinction is relevant when determining an appropriate MGMTpm cut-off, as IDHmut grade 4 astrocytomas—commonly included in studies prior to the WHO 2021 classification —are associated with a more favourable prognosis [37, 38]. Thus, our findings may be more directly applicable to contemporary clinical practice.

It is worth noting that MGMTpm testing in several Swedish pathology departments has lately shifted from Therascreen pyrosequencing to the recently introduced PentaBase Epidirect qPCR-based method, following withdrawal of the former from the market. While Epidirect offers technical advantages, including omission of bisulfite pretreatment, its clinical validation remains limited and is based on relatively small cohorts, and currently defined cut-offs may not yet be robust [39]. If widely adopted as the replacement for MGMTpm testing, further validation of the method and definition of clinically relevant cut-offs will be essential to ensure reliable interpretation in patients with GBM.

In conclusion, our findings support a simple three-tier classification of MGMTpm for the Therascreen Kit investigating CpGs 76–79. In our cohort treated with concomitant RT/TMZ, a threshold of ≤ 8% identifies a subgroup with poor survival consistent with a truly unmethylated phenotype, while values ≥ 11% correspond to a clearly methylated group with substantially improved outcomes. The intermediate range represents a gray zone, in which individualized treatment decisions are warranted. Overall, our results emphasize the importance of assay-specific validation of MGMTpm cut-offs, integrating both biological distribution and clinical outcome data, to ensure robust and clinically meaningful stratification in patients with GBM.

## Supplementary Information

Below is the link to the electronic supplementary material.

Supplementary Material 1

## Data Availability

The datasets generated and/or analyzed during the current study are available from the corresponding author on reasonable request.
