# Supplementary material for: MGMT promoter methylation status for glioblastoma: defining the clinically relevant cut-off value for pyrosequencing
Source: J Neurooncol. 2026 May 20;178(1):12. doi: 10.1007/s11060-026-05633-0 (PMC13190539; doi:10.1007/s11060-026-05633-0)
Supplement: Supplementary file 1 — Supplementary Material 1 [file 11060_2026_5633_MOESM1_ESM.pdf]

MGMT Promoter Methylation Status for Glioblastoma: Defining the Clinically Relevant Cut-off Value for pyrosequencing

Journal of Neuro-Oncology

Nathalie Skarin, Martin Hallbeck, Katja Werlenius, Zdenek Rohan, Maria Sandström, Eszter Turanyi, David Löfgren, Håkan Johansson, Michael Strandéus, Björn Tavelin, Annika Malmström

Corresponding author: Annika Malmström, Clinical Department of Geriatrics and Palliative Medicine in Linköping, Region Östergötland, Linköping, and Department of Biomedical and Clinical Sciences, Linköping University, Linköping, Sweden; annika.malmstrom@regionostergotland.se

Supplementary material

Supplementary information on statistical analysis

We determined a supervised (i.e. survival-informed) cut-off by modelling survival time using an accelerated failure time (AFT) model with a log-logistic distribution [23]. An AFT model was chosen to allow direct comparison of survival time ratios and optimize threshold discrimination. The model contained the dichotomized methylation status (i.e. methylated or unmethylated) with adjustments for prognostic factors (age, extent of surgery, and preopPS) incorporated as independent linear effects. The final cut-off was determined by varying the cut-off from 1 to 25% in increments of 1%; for each candidate cut-off, the model was refitted, its predictive performance was evaluated using 5-fold cross-validation, and the cut-off from the model with the highest concordance index was selected as optimal. Briefly, the concordance index measures predictive performance by considering all pairs of patients whose survival times can be ordered [24]. If the patient with the longer observed survival time also hade the longer predicted survival time, the pair is said to be concordant. The concordance index is the proportion of comparable pairs that are concordant and can be interpreted as the probability that the model can correctly rank which of two patients will survive longer.

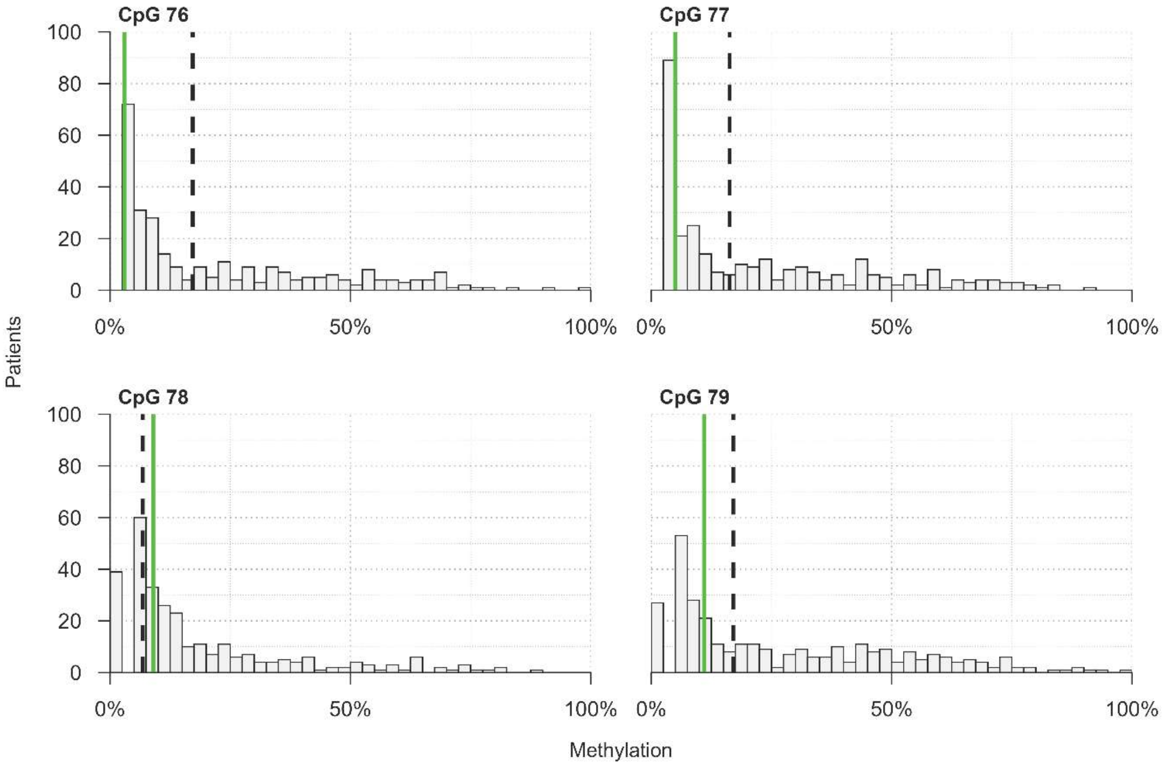

Supplementary Fig. 1 Distributions for each individual CpG of all 451 patients. Dashed black and solid green lines are explained in Supplementary Figures 2 and 3, respectively

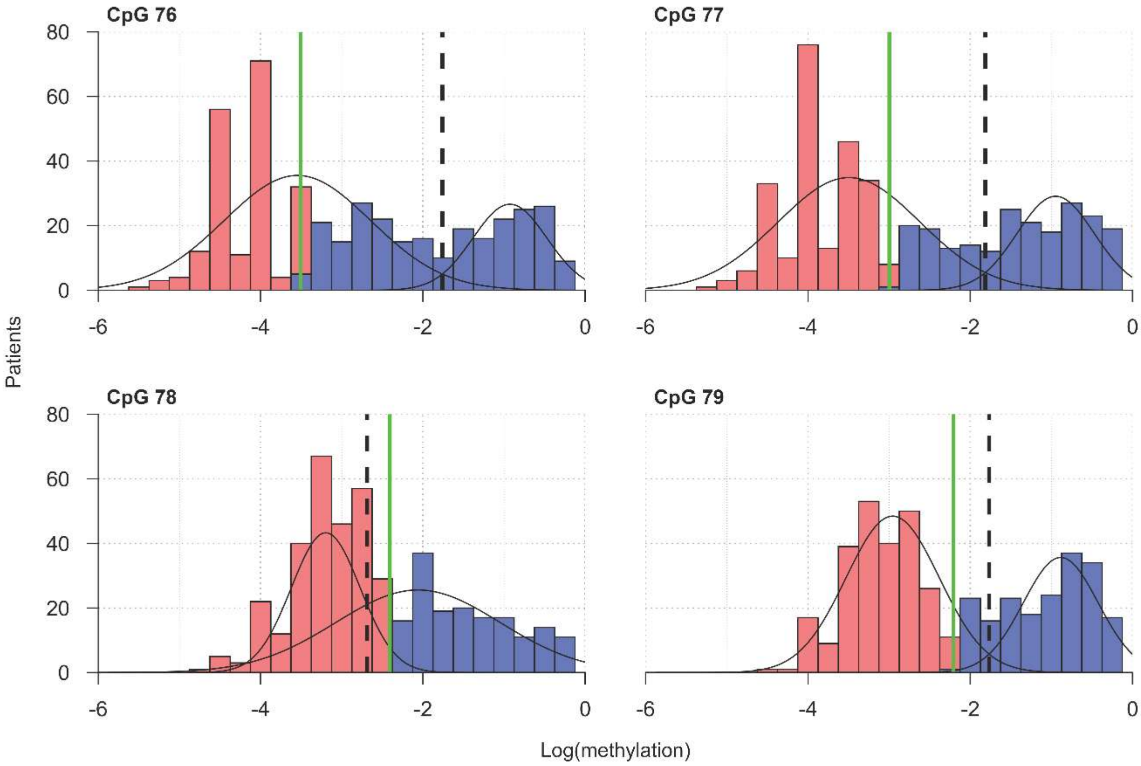

Supplementary Fig. 2 Bimodal normal mixture model of each individual CpG, with unsupervised cut-off (dashed black line) determined as the intersection point of the two distributions. Blue bars represent patients with methylated MGMT, and red bars represent patients with unmethylated MGMT. During the development of bimodal mixture models for individual CpG sites, significant instability in the solutions was observed for two of the sites. When fitting mixture models, such instability typically indicates that the data are compatible with multiple plausible solutions and that there is insufficient evidence to clearly favour one over another. Consequently, the fitted model may be sensitive to the choice of initialization values. To investigate this instability, each model was run 400 times using different initializations. All runs converged on the same optimal cut-off (i.e., intersection point) for mean methylation, CpG 76, and CpG 79, while both CpG 77 and CpG 78 exhibited multiple solutions in more than one-third of the runs. These findings indicate that thresholds derived from the bimodal mixture models for CpG 77 and CpG 78 should be interpreted with caution, given their sensitivity to initialization.

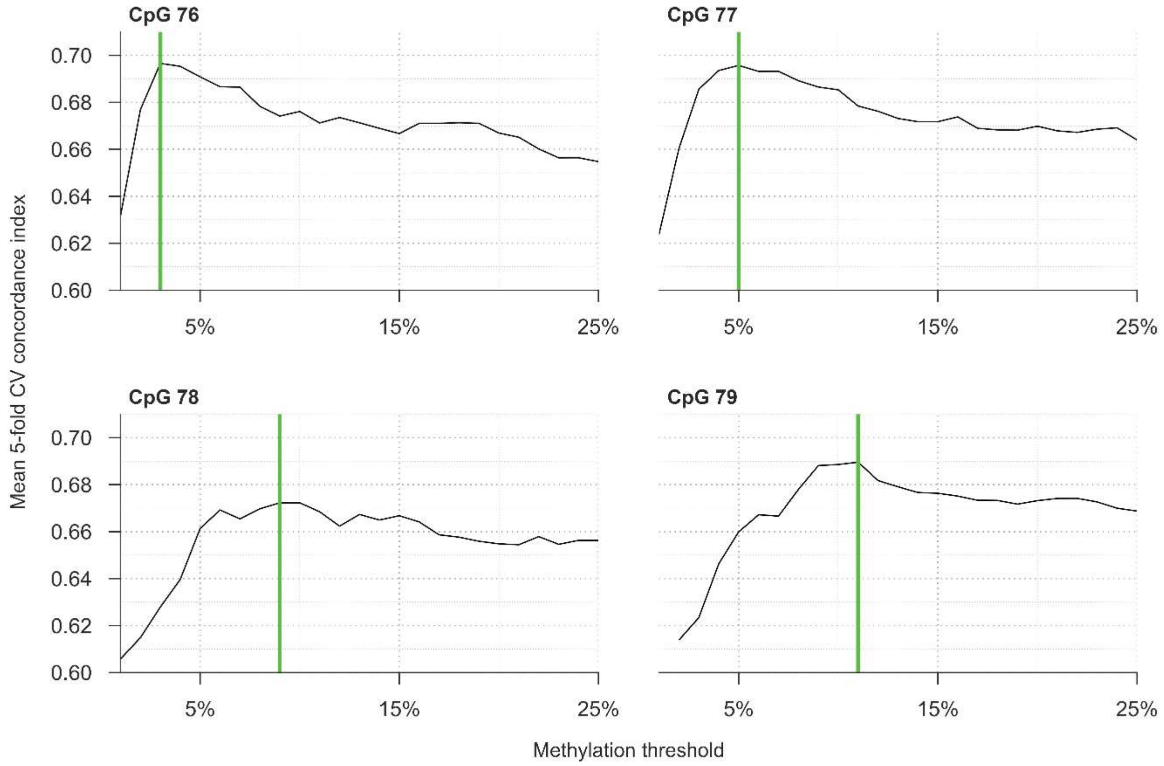

Supplementary Fig. 3 Cut-off supervised by OS and adjusted for prognostic factors: age, extent of resection and preoperative ECOG performance status (solid green line), determined through 5-fold cross-validation with the highest concordance index corresponding to the best performing AFT (accelerated failure time) model. Compared to mean methylation, no single CpG had significantly superior predictive performance, although differences were small (concordance index differences: -2.13–0.21%)

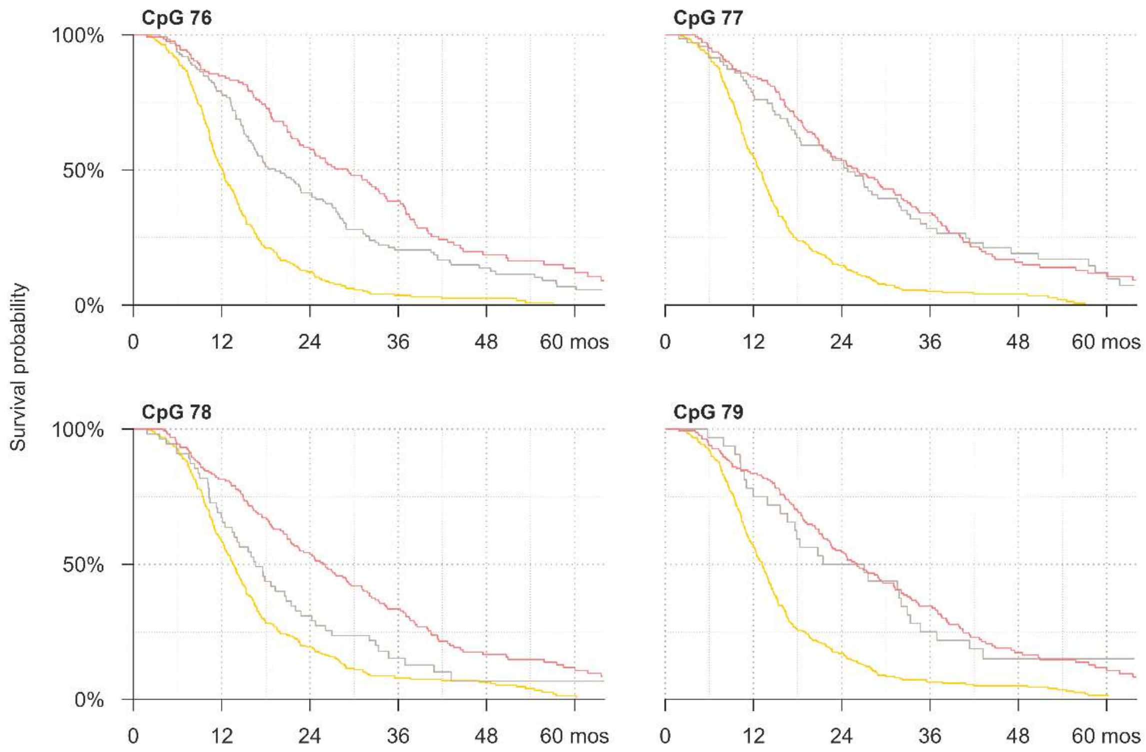

Supplementary Fig. 4 Kaplan-Meier curves demonstrating survival for patients with truly uMGMT, MGMTpm in the gray zone and mMGMT GBM. Across all CpGs, survival differed significantly among the three groups (log-rank tests; all p < 0.001).

Supplementary Table 1 Clinical outcomes based on supervised methylation status

Parameter	Truly unmethylated MGMT	Gray zone	Methylated MGMT
**Number of patients, n (%)**	248 (54.9%)	15 (3.3%)	188 (41.8%)
**12 month overall survival, n (%)**	137 (55.2%)	10 (66.7%)	158 (84.0%)
**24 month overall survival, n (%)**	43 (17.3%)	4 (26.7%)	104 (55.3%)
**60 month overall survival, n (%)**	2 (0.8%)	0 (0%)	13 (69%)
**Median overall survival, months (range)**	12.9 (2.1–60.2)	17.6 (5.7–34.7)	26.6 (1.8–78.9)

Supplementary Table 2 Parameter estimates from the multivariate AFT model of overall survival.

Variable	Exponentiated estimate*	CI 2.5%	CI 97.5%	p-value
**Median baseline survival time (months)[a]**	16.9	14.8	19.3	< 0.005
**Methylation> threshold**	1.9	1.7	2.2	< 0.005
**preopPS [b]: 1**	1.0	0.8	1.1	0.83
**preopPS: ≥2**	0.9	0.8	1.0	0.13
**Extent of resection[c]: Near-total resection (<1 cm³ residual tumor)**	0.9	0.7	1.0	0.04
**Extent of resection: Subtotal resection**	0.7	0.6	0.8	< 0.005
**Extent of resection: Biopsy**	0.5	0.4	0.6	< 0.005
**Age at surgery (per SD increase)**	0.9	0.8	0.9	< 0.005

[a]The median baseline survival time is the expected median survival time when all covariates are at their reference values, i.e., for a patient of average age at operation for the dataset (60.9 years) with unmethylated MGMT, preoperative performance status 0, and gross total resection. [b]Preoperative performance status measured by the ECOG performance status scale. [c] Extent of resection was primarily determined based on postoperative MRI. In cases where imaging assessment was unavailable or inconclusive, surgical intent was used.

*The exponentiated estimates are time ratios, i.e. the factor by which the median baseline survival time is multiplied to account for a change in category or for one standard deviation increase in age at surgery (10.4 years). Values greater than one indicate longer survival times, values less than 1 indicate shorter survival times.
